# FRAX-based fracture probabilities in South Africa

**DOI:** 10.1007/s11657-021-00905-w

**Published:** 2021-03-01

**Authors:** Helena Johansson, Sapna S. Dela, Bilkish Cassim, Farhanah Paruk, Susan L. Brown, Magda Conradie, Nicholas C. Harvey, Johannes D. Jordaan, Asgar A. Kalla, Enwu Liu, Mattias Lorentzon, Mkhululi Lukhele, Eugene V. McCloskey, Ozayr Mohamed, Pariva Chutterpaul, Liesbeth Vandenput, John A. Kanis

**Affiliations:** 1 Mary McKillop Institute for Health Research, Australian Catholic University, Melbourne, Australia; 2 Centre for Metabolic Bone Diseases, University of Sheffield Medical School, Beech Hill Road, S10 2RX, Sheffield, UK; 3 Department of Internal Medicine, Edendale Hospital, School of Clinical Medicine (SCM), University of KwaZulu-Natal, Durban, South Africa; 4 Department of Geriatrics, School of Clinical Medicine (SCM), College of Health Sciences, University of KwaZulu-Natal, Durban, South Africa; 5 Division of Internal Medicine, School of Clinical Medicine, College of Health Sciences, University of KwaZulu-Natal, Durban, South Africa; 6 Department of Medicine, Mahathma Gandhi Memorial Hospital, Durban, South Africa; 7 Division of Endocrinology, University of Stellenbosch, Stellenbosch, South Africa; 8 MRC Lifecourse Epidemiology Unit, University of Southampton, Southampton, UK; 9 Division of Orthopaedics, University of Stellenbosch, Stellenbosch, South Africa; 10 Division of Rheumatology, Faculty of Health Sciences, University of Cape Town, Cape Town, South Africa; 11 Geriatric Medicine, Institute of Medicine, University of Gothenburg, Gothenburg, Sweden; 12 Department of Orthopaedics, University of Witwatersrand, Witwatersrand, South Africa; 13 Mellanby Centre for bone research, Department of Oncology and Metabolism, University of Sheffield, Sheffield, UK; 14 Discipline of Public Health Medicine, SCM, College of Health Sciences, UKZN, Durban, South Africa; 15 Department of Internal Medicine and Clinical Nutrition, Institute of Medicine, Sahlgrenska Academy, University of Gothenburg, Gothenburg, Sweden

**Keywords:** FRAX, Fracture probability, Osteoporosis, Epidemiology, Hip fracture, South Africa

## Abstract

**Summary:**

The hip fracture rates in South Africa were used to create ethnic-specific FRAX® models to facilitate fracture risk assessment.

**Introduction:**

The aim of this study was to develop FRAX models to compute the 10-year probability of hip fracture and major osteoporotic fracture and assess their potential clinical application.

**Methods:**

Age- and sex-specific incidence of hip fracture and national mortality rates were incorporated into a FRAX model for the White, Black African, Coloured and Indian population of South Africa. Age-specific 10-year probabilities of a major osteoporotic fracture were calculated in women to determine fracture probabilities at a femoral neck *T* score of -2.5 SD, or those equivalent to a woman with a prior fragility fracture. Fracture probabilities were compared with those from selected countries.

**Results:**

Probabilities were consistently higher in Indian than in Coloured men and women, in turn, higher than in Black South Africans. For White South Africans, probabilities were lower than in Indians at young ages up to the age of about 80 years. When a BMD *T* score of −2.5 SD was used as an intervention threshold, FRAX probabilities in women age 50 years were approximately 2-fold higher than in women of the same age but with an average BMD and no risk factors. The increment in risk associated with the BMD threshold decreased progressively with age such that, at the age of 80 years or more, a *T* score of −2.5 SD was no longer a risk factor. Probabilities equivalent to women with a previous fracture rose with age and identified women at increased risk at all ages.

**Conclusions:**

These FRAX models should enhance accuracy of determining fracture probability amongst the South African population and help guide decisions about treatment.

## Introduction

Osteoporosis is a common, chronic and costly condition; its clinical consequence is fracture that in turn is a major cause of disability and death [1]. Disability due to osteoporosis is greater than that caused by any single cancer, with the exception of lung cancer and comparable or greater than that lost to a variety of chronic noncommunicable diseases, such as rheumatoid arthritis, asthma and high blood pressure-related heart disease [2, 3]. A wide variety of treatments is available that favourably affect bone mass and thereby decrease the risk of fractures associated with osteoporosis [4]. The use of such interventions by health care practitioners is assisted by instruments that assess patients’ fracture risk to optimise clinical decisions about prevention and treatment.

The most widely used web-based tool FRAX® (https://www.sheffield.ac.uk/FRAX/) meets these requirements and computes the 10-year probability of fragility fractures based on several common clinical risk factors and, optionally a DXA scan result [5, 6]. FRAX models are available for 73 countries in 2020 covering more than 80% of the world population at risk [7] and have been incorporated into more than 100 guidelines worldwide [8].

The availability of FRAX has stimulated studies of the incidence of hip fractures that have been undertaken for the generation of new FRAX models. Specific examples include Brazil, Mexico, Turkey [9] and several countries of Eastern Europe [10–14]. Recently, ethnic and gender-specific incidence rates for hip fractures have been reported for South Africa [15]. This report describes the characteristics of FRAX-based fracture probability models derived from the risks of hip fracture and death in each ethnic group of South Africa.

## Methods

The Republic of South Africa is the southernmost country in Africa with a population of 57.429 million and an area of 1,221,037 square kilometres (758,717 square miles). Approximately 80% of South Africans are of African ancestry (Black) and the remaining population comprises mainly European (White), Indian and multiracial ancestry (Coloured). The term ‘coloured’ can be offensive in some parts of the world including the UK (https://www.bbc.co.uk/news/newsbeat-54888197). There are, however, places in the world where ‘coloured’ is used without offence - for example in South Africa, where it refers to people who have multiple heritages. Indeed, Statistics South Africa asks people to self-identify in terms of racial population groups, namely, Black South African, White South African, Coloured South African, Asian South African and other/unspecified [16]. Given that this paper describes ethnic-specific characteristics in South Africa, we have retained the official terms.

Data on the incidence of hip fracture have been previously published [15]. In brief, incidence was studied prospectively in 94 hospitals in eight geographically defined districts of three provinces. The provinces Gauteng, Western Cape and KwaZulu-Natal were chosen to optimise representation of the major ethnic groups, Black, White, Indian and Coloured. Low energy hip fractures were documented from April 1, 2017, to March 31, 2018 in all individuals age 40 years or more. The catchment population was estimated at 4,034,153, representing 29·5% of the total population age 40 years or over. Crude incidence was the highest in the White (129·9 per 100,000) and Indian populations (111·7 per 100,000) and lower in the Coloured (58·2 per 100,000) and Black populations (37·9 per 100,000).

The data on hip fracture were used to construct four FRAX models, one for each ethnic group. For other major osteoporotic fractures (clinical spine, forearm and humeral fractures), it was assumed that the age- and sex-specific ratios of these fractures to hip fracture risk were comparable with those found in Sweden [17]. This assumption has been used for many of the FRAX models with incomplete epidemiological information on non-hip fractures.

The development and validation of FRAX have been extensively described [4, 5]. The risk factors used were based on a systematic set of meta-analyses of population-based cohorts worldwide and validated in independent cohorts with over 1 million patient-years of follow-up. The construct of the FRAX model for South Africa retained the beta coefficients of the risk factors in the original FRAX model with the incidence rates of hip fracture and mortality rates for South Africa. Mortality rates, which are traditionally supplied by the World Health Organization, were not available by ethnicity but were made available through Statistics South Africa [16].

In South Africa, the current threshold for treatments is based on BMD measurements using DXA with a threshold for reimbursement set at a *T* score of −2.5 SD [18]. The South African models were used to calculate the ten-year probabilities of a major osteoporotic fracture by age (in 5-year increments from the age of 50 to 90 years) in women at the threshold of osteoporosis (*T* score = −2.5 SD). As for all FRAX models, the *T* score was based on the NHANES III as a reference for BMD at the femoral neck in Caucasian women aged 20–29 years [19]. Women were assumed to have no other clinical risk factors that might contribute to fracture probability. The calculation of fracture probability was made at a body mass index (BMI) of 25 kg/m^2^. Changes in BMI have little effect on predictive value for fracture risk assessment in the presence of BMD [20].

Since treatment is commonly recommended in women with a previous fragility fracture, a second intervention threshold was calculated over the same age increments in women with a prior fracture but no other clinical risk factors using the ethnic-specific FRAX tools, without BMD and a BMI set at 25 kg/m^2^.

In order to compare hip fracture probabilities with those of other regions of the world, the remaining lifetime probability of hip fracture from the age of 50 years was calculated for men and women, as described previously [21]. In the present analysis, values for South Africa were compared with those of Abu Dhabi, Bulgaria, Canada, China (Hong Kong), Denmark, Finland, France, Germany, Greece, Hungary, Iran, Kazakhstan, Kuwait, Mexico, Moldova, Morocco, Netherlands, Poland, Portugal, Romania, Russia, Singapore (Indian), Spain, Sweden, Tunisia, Turkey, the UK, Ukraine and the USA (African and Caucasian). Hazard functions for fracture and death were those used in the relevant FRAX models.

## Results

**Fig. 1:**
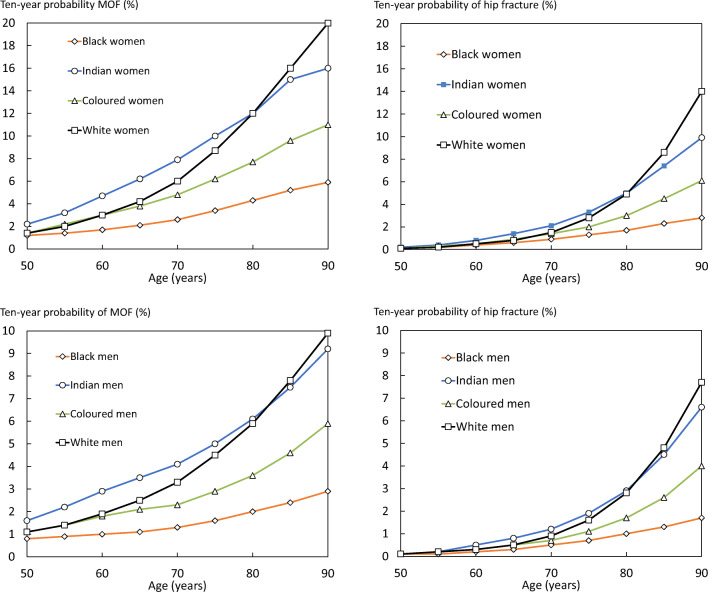
Ten-year probability of major osteoporotic fracture (MOF) and hip fracture (%) in men and in women by ethnic group (no clinical risk factors, BMI of 25 kg/m^2^ and no BMD entered)

In women with no clinical risk factors, 10-year fracture probabilities rose with age for all ethnicities (Fig. 1). Age-dependent increases were more marked in women than in men. Probabilities were consistently higher in Indian than in Coloured men and women, in turn, higher than in Black South Africans. For White South Africans, probabilities were lower than in Indians at young ages up to the age of about 80 years after which probabilities exceeded all other ethnicities.

### *T* score threshold

The clinical significance of a given *T* score varied by ethnicity (Table 1). For example, in women age 65 years at the threshold for osteoporosis (femoral neck *T* score = −2.5), the 10-year probability of a major osteoporotic fracture ranged from 3.3% (African) to 9.2% (Indian) with intermediate values for the White and Coloured women (6.3 and 5.7%, respectively).

**Table 1:** Ten-year probability of a major osteoporotic fracture by age and ethnic group in women with no clinical risk factors (CRF), a prior fragility fracture (Prior Fx) and a femoral neck *T* score of −2.5 SD with no other risk factors. Body mass index set at 25 kg/m^2^

	White			Indian			Coloured			Black		
Age	No CRF	Prior Fx	T-2.5	No CRF	Prior Fx	T-2.5	No CRF	Prior Fx	T-2.5	No CRF	Prior Fx	T-2.5
50	1.4	3.0	2.8	2.2	4.7	4.3	1.4	3.1	2.8	1.2	2.6	2.4
55	2.0	4.3	3.7	3.2	6.8	5.9	2.2	4.6	3.9	1.4	3.1	2.8
60	3.0	6.2	5.0	4.7	9.7	7.9	3.0	6.3	5.0	1.7	3.7	3.1
65	4.2	8.4	6.3	6.2	12	9.2	3.8	7.6	5.7	2.1	4.3	3.3
70	6.0	11	7.9	7.9	15	10	4.8	9.1	6.2	2.6	5.1	3.5
75	8.7	15	10	10	18	11	6.2	11	6.9	3.4	6.2	3.9
80	12	20	12	12	20	12	7.7	13	7.4	4.3	7.2	4.1
85	16	25	13	15	23	12	9.6	16	7.8	5.2	8.7	4.3
90	20	30	14	16	25	11	11	18	7.8	5.9	9.8	4.2

The significance of a given *T* score also varied by age. In women age 50 years at the threshold of osteoporosis (*T* score = −2.5 SD), fracture probability was approximately 2-fold higher than in women of the same age but with an average BMD and no risk factors, irrespective of ethnicity (Table 1). As expected, the probability at the osteoporosis threshold increased with age; for example, in white women, the 10-year fracture probability rose progressively from 2.8% at the age of 50 years to 14% at the age of 90 years. However, the probability ratio between those at the osteoporosis threshold and women without clinical risk factors decreased with age and, at the age of 60, 70 and 80 years, were 1.7, 1.3 and 1.0, respectively.

The fracture probabilities equivalent to women with a previous fragility fracture are shown in Table 1. The probabilities rose with age. Fracture probabilities using this threshold were consistently higher than in women with no clinical risk factors, an effect that contrasted with the waning effect of *T* score with age.

**Fig. 2:**
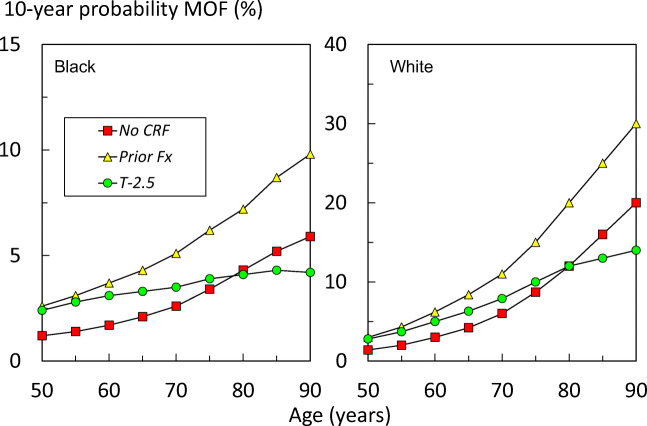
Ten-year probabilities of a major osteoporotic fracture (MOF; hip, clinical spine, humerus and forearm) calculated with the South African FRAX model for African and White women with no clinical risk factors (CRF), a prior fragility fracture (Prior Fx) and a femoral neck *T* score of -2.5 SD with no other risk factors.

The phenomenon is illustrated for the White and African models representing the higher and lower probability models (Fig. 2).

Lifetime probabilities for hip fracture are shown in Table 2. South African White women had fracture probabilities comparable to those in Northern Europe and substantially higher than their ancestral equivalents (UK, Netherlands and Germany). In contrast, lifetime probabilities of South African Indians were remarkably similar to those of Indians from Singapore. The differences in probabilities between the USA and South African Blacks were not marked with slightly higher rates in the former. South African Blacks and Coloureds had probabilities higher than those available for Saharan Africa (Tunisia, Morocco).

**Table 2:** Life-time probability of hip fracture in the South African population from the age of 50 years compared with selected countries, ranked in descending order of lifetime probability in women

Country	Life-time probability from 50 years (%)	
	Women	Men
Sweden	25.6	11.0
South Africa (White)^1^	23.4	7.7
Denmark	23.0	11.3
France	19.3	5.9
China (Hong Kong)	17.7	7.6
USA (Caucasian)	16.1	7.5
Turkey	15.9	3.6
Canada	15.5	5.8
Greece	15.4	6.8
UK	14.4	5.0
Germany	14.2	5.3
Portugal	13.7	4.8
Finland	12.9	6.0
Kazakhstan	12.6	6.0
Spain	12.6	4.2
Netherlands	12.5	5.4
Singapore (Indian)	12.5	5.2
South Africa (Indian)^1^	12.1	4.6
Bulgaria	11.2	4.4
Hungary	10.8	4.2
Mexico	10.6	5.0
Poland	10.1	4.2
Moldova	9.3	5.7
Kuwait	9.2	7.6
Abu Dhabi	8.9	8.1
Iran	8.3	5.5
Russia	7.7	3.8
Romania	7.0	3.8
South Africa (Coloured)^1^	7.0	2.7
USA (African)	5.9	2.7
Ukraine	5.6	2.9
South Africa (Black)^1^	4.5	1.9
Morocco	4.1	3.1
Tunisia	0.7	0.7

^1^This study

## Discussion

This study documented the 10-year probabilities of hip fracture and major osteoporotic fracture in the Republic of South Africa. As expected from the incidence of hip fracture, there were marked differences in the fracture probability between ethnic groups. The differences justify the use of ethnic-specific FRAX models as found also for the USA and Singapore versions of FRAX. On an international basis, FRAX for the Indian population belongs to the moderate-risk countries for MOF probability for men and women. The Coloured and Black community are in the low risk category and Whites lie in between [22].

In this study, we examined two scenarios for the assessment of women at high fracture risk based on the four FRAX tools for South Africa. The first related to a femoral neck *T* score threshold of −2.5 SD commonly used as an intervention threshold and included in the guidance for South Africa [18]. A fixed threshold based on the *T* score of −2.5 SD has the advantage of simplicity and universality, but it also has important limitations. As shown in this study, fracture probability differed markedly between ethnic groups for any given *T* score. Additionally, the increase in risk associated with a *T* score threshold of −2.5 SD diminished with advancing age. Indeed, from the age of 80 years, a *T* score of −2.5 SD was protective, in the sense that the fracture probability was lower than that of the general population (with no clinical risk factors) at that age. Thus, the BMD criterion for intervention using a fixed *T* score becomes less and less appropriate with advancing age [23–25]. The situation arises because the *T* score of the general population decreases with age so that the average *T* score in the elderly is less than −2.5. These considerations suggest that intervention thresholds based on the *T* score alone do not effectively target treatment.

The second scenario examined was the impact of a prior fracture on fracture probabilities. Although fracture probabilities varied between ethnic models, the increase in risk associated with a prior fracture was not attenuated with age (see Fig. 2). This supports the recommendation of very many practice guidelines that women with a prior fragility fracture should be considered for treatment [8]. If women with a prior fragility fracture merit intervention, then women with a fracture probability that equals or exceeds that of women with a prior fracture should also be eligible for treatment. This forms the basis of FRAX based intervention thresholds developed in many countries [8, 26]. These considerations indicate that the gateway to fracture risk assessment is more logically based on fracture probability than on BMD. The fracture probability equivalent to a woman with a prior fracture has been used as an intervention threshold in more than 30 countries. If the same threshold were applied to South Africa, then intervention would be recommended with a probability of a major osteoporotic fracture that varied between 2.6 and 30 % depending on age and ethnicity. The impact of such thresholds or alternative thresholds will require further study.

As was the case for South Africa, a majority of countries that have a FRAX model do not have robust information on the risk of other major osteoporotic fractures. In the absence of such information, FRAX models assume that the age- and sex-specific pattern of these fractures is similar to that observed in Malmo, Sweden [17]. This assumption has been shown to be safe in studies reported from Canada [27], Iceland [28], the USA [29], the UK [30], Australia [31] and Moldova [13], despite marked differences in incidence between these countries [23]. This commonality of pattern is supported by register studies which indicate that, in those regions where hip fracture rates are high, so too is the risk of forearm fracture and spine fractures (requiring hospital admission) [32, 33].

The incidence of hip fracture was used to create FRAX tools to compute the 10-year probabilities of hip and major osteoporotic fracture in South Africa, now available on the FRAX web site (https://www.sheffield.ac.uk/FRAX/tool.aspx?country=79). Although FRAX tools are available in all continents, this is a first for sub-Saharan Africa. Thus, there are no neighbouring countries available to make comparisons. It is notable, however, that fracture probabilities in Indians from South Africa were remarkably similar to those found in Indians from Singapore. In the case of Black Africans, probabilities were somewhat lower in those from South Africa than those from the USA. It is of interest that the hip fracture incidence in the Black community was similar to a recent but smaller study in South Africa [34].

There are a number of additional limitations to this study. With regard to fracture incidence, this was based on approximately one third of the total population, albeit from eight geographically defined districts of three provinces. Therefore, the applicability of this regional estimate to the entire country is an assumption that we were unable to test.

In addition to large variations in fracture rates around the world, fracture rates may vary within countries over and above ethnic-specific differences [35–37]. Up to 2-fold differences in hip fracture incidence have been reported using common methodology with the higher rates in urban communities including Croatia [38], Switzerland [39], Norway [40], Argentina [41] and Turkey [42].

It is relevant, however, that accuracy errors have little impact on the rank order with which the FRAX tool categorises risk in a given population [11, 43, 44] but they do change the absolute number generated and thus have implications where treatment guidelines are based on cost-effectiveness or the economic burden of disease.

In summary, four ethnic-specific FRAX models have been created for the Republic of South Africa that are based on a regional population-based estimate of the incidence of hip fracture. The model should enhance accuracy of determining fracture probability amongst the South African population and help to guide decisions about treatment.
